# Ge nanoparticles in SiO_2_ for near infrared photodetectors with high performance

**DOI:** 10.1038/s41598-019-46711-w

**Published:** 2019-07-16

**Authors:** Ionel Stavarache, Valentin Serban Teodorescu, Petronela Prepelita, Constantin Logofatu, Magdalena Lidia Ciurea

**Affiliations:** 1 National Institute of Materials Physics, 405A Atomistilor Street, 077125 Magurele, Ilfov Romania; 2 National Institute for Laser, Plasma and Radiation Physics, 409 Atomistilor Street, 077125 Magurele, Ilfov Romania; 3 Academy of Romanian Scientists, 050094 Bucharest, Romania

**Keywords:** Sensors and biosensors, Electronic properties and materials, Composites

## Abstract

In this work we prepared films of amorphous germanium nanoparticles embedded in SiO_2_ deposited by magnetron sputtering on Si and quartz heated substrates at 300, 400 and 500 °C. Structure, morphology, optical, electrical and photoconduction properties of all films were investigated. The Ge concentration in the depth of the films is strongly dependent on the deposition temperature. In the films deposited at 300 °C, the Ge content is constant in the depth, while films deposited at 500 °C show a significant decrease of Ge content from interface of the film with substrate towards the film free surface. From the absorption curves we obtained the Ge band gap of 1.39 eV for 300 °C deposited films and 1.44 eV for the films deposited at 500 °C. The photocurrents are higher with more than one order of magnitude than the dark ones. The photocurrent spectra present different cutoff wavelengths depending on the deposition temperature, i.e. 1325 nm for 300 °C and 1267 nm for 500 °C. These films present good responsivities of 2.42 AW^−1^ (52 *μ*W incident power) at 300 °C and 0.69 AW^−1^ (57 mW) at 500 °C and high internal quantum efficiency of ∼445% for 300 °C and ∼118% for 500 °C.

## Introduction

In the last decade, many research groups have paid attention on amorphous germanium nanoparticles (Ge-NPs) embedded in different oxide matrices because of their attractive electrical and optical properties which are suitable for different applications like photo-detectors^1–3^, solar cells^4^, light-emitting diodes^5^, memory devices^6^, MOSFET transistors^7,8^ and lithium-ion batteries with high charge-discharge rate^9,10^. This effort has the main aim to extend the sensitivity domain of photodetectors toward near infrared (NIR) and therefore to develop the optoelectronics in this wavelength range as Si based photodetectors are usually limited to 1.1–1.2 *μ*m. Ge is the main candidate that has attracted attention for Si replacement because Ge has a higher carrier mobility than Si^11^. Also, Ge is a nontoxic material, biocompatible, cheap and (electro)chemically stable. Though Ge has crystalline structure similar to that of Si, Ge has different electronic properties such as smaller band gap (0.66 eV in respect to 1.1 eV) and the excitonic Bohr radius is bigger (about 24 nm) than that of Si (about 5 nm)^12^. These characteristics allow for a stronger quantum confinement effect compared to Si^13^. The ability to control the Ge-NPs characteristics, such as particle shape, size and density will facilitate a better control of their optical and electrical properties^14–17^ that are very important for applications. The optical and electrical processes in the Ge-NPs based materials can be also strongly influenced by the mid-gap states and defects located at the interface with the matrix^18^, degree of crystallization^19^ as well as the nature of the surrounding matrix^18,20,21^. For obtaining Ge-NPs embedded in SiO_2_ (Ge-NPs:SiO_2_), different deposition methods have been used like chemical vapor depositions^22^, laser ablation^23^, implantation^24^, sputtering^25^, hybrid and colloidal methods^26,27^, the deposition being followed or not by temperature annealing. Besides these, an important step to obtain high quality films is related to the optimal conditions used for Ge-NPs formation as Ge easily oxidizes. In presence of oxygen (residual or from the matrix) and depending on environment conditions Ge can form two types of oxides GeO and GeO_2_. Since 1973, Frantsuzov *et al*. proves that Ge losses from films can take place at relatively low temperature around 360 °C through the reaction between clean Ge surface and oxygen molecules^28^. The studies conducted by Ramana *et al*. reveal that GeO_2_ has a great thermal stability and an optical bandgap of 5.7 eV^29^. Depending on the targeted goal, Ge-NPs can be obtained by choosing different pathways. For example, the temperature necessary for Ge-NPs formation is influenced by the host matrix and if the annealing is performed in a furnace or in a rapid thermal processor. So, Ge-NPs are formed at about 310 °C^30^ in indium tin oxide (ITO), in the range of 800–1000 °C^31,32^ in Al_2_O_3_ or above 700 °C in ZrO_2_ matrices^33^. Structures based on Ge-NPs deposited on glass or flexible substrates can be obtained using or not a thermal treatment during the deposition process^34–36^.

In this paper, we investigate the films formed of Ge-NPs:SiO_2_ with high photoresponse for visible (VIS)—NIR detection. In this aim, we study the effect of the deposition temperature on the morphology and photo-electrical properties of Ge-NPs:SiO_2_ thin films. The films were deposited on Si and quartz substrates at temperatures of 300, 400 and 500 °C maintaining the others deposition parameters at constant values. The morphology of films is studied by transmission electron microscopy (TEM), x-Ray photoelectron spectroscopy (XPS) and corelated with their composition. On these films, optical (transmittance and reflectance), and electrical measurements (current density–voltage (*J*–*V*) characteristics) in dark and under illumination and photocurrent spectra were performed. This approach brings important contribution to the effort of structuring Ge-NPs in SiO_2_ thin films at lower temperature (during deposition) in the aim of using the performance of these materials towards integrated optoelectronics.

## Results and Discussion

### Morphology and crystalline structure

**Figure 1:**
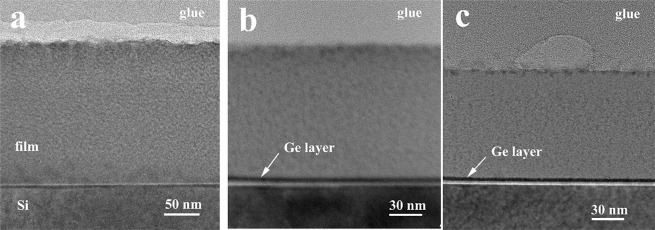
Low magnification XTEM images of the films deposited at 300 °C (**a**); 400 °C (**b**) and 500 °C (**c**).

Low magnification XTEM images of films deposited on Si substrates at 300 °C (a), 400 °C (b) and 500 °C (c) are shown in Fig. 1. The thickness and the morphology of the films are different. As it can be observed, the deposited films at 300 °C have a thickness of 200 nm, 123 nm at 400 °C and 100 nm at 500 °C, in good agreement to the deposition rates (see Methods Section). The film thicknesses are due only to the substrate temperatures, the rest parameters (pressure, applied power, deposition time) were kept constant.

**Figure 2:**
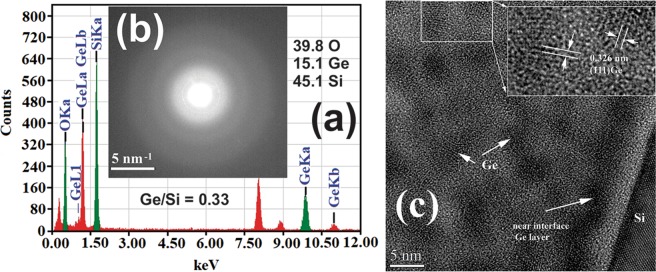
(**a**) EDX spectrum of the GeSiO film deposited at 300 °C; (**b**) SAED measured on the film deposited at 300 °C, showing the amorphous structure of the film; (**c**) HRTEM image of Ge-NPs:SiO_2_ film deposited at 500 °C; few Ge-NPs are crystallized, but the majority remain amorphous (inset).

Also, one can observe that a Ge layer appears near the Si interface in the case of films deposited at 400 °C and 500 °C and a small agglomeration for films deposited at 300 °C. The atomic Ge/Si ratio in the film deposited at 300 °C is 0.33 as revealed by the EDX measurements (Fig. 2a). Selected area electron diffraction (SAED) also, shows that GeSiO film is amorphous (Fig. 2b). Figure 2c shows the image taken from the Ge-NPs:SiO_2_ film deposited at 500 °C. The EDX spectrum in Fig. 2a was collected in an area of 50 nm diameter in the middle of the GeSiO_2_ film thickness (XTEM specimen), in order to have a correct average ratio between the Ge and Si atoms in the film. Ge/Si ratio is 0.33 and was calculated by using the SiK and GeK lines of the EDX spectrum.

HRTEM image obtained on Ge-NPs:SiO_2_ films deposited at 500 °C is presented in Fig. 2c–inset and reveals that few of the Ge-NPs:SiO_2_ matrix are crystallized, but the majority remain amorphous. In this image, it is quite clear that the interface layer is formed by dense Ge-NPs. It is pretty sure that in this layer we have both metallic Ge and GeO_*x*_ species. The problem is that in this layer (film deposited at 500 °C) lattice fringes of Ge crystallites were not found, as in the case of Ge-NPs present in the film volume. This suggests that here the NPs contain more GeO_*x*_ species (in good agreement with XPS) than in the rest of film, and this can hinder crystallization. For the moment, we have not an explanation for the formation of this interface layer, which appears in the case of the Ge-NPs:SiO_2_ films deposited on heated substrate but also in the other Ge-NPs:SiO_2_ films deposited on room temperature (RT) substrates and then annealed^37^. In the Ge-NPs:SiO_2_ film deposited at 500 °C, the real size of the Ge crystallites can be measured using the lattice fringes number for each crystallite. In the most cases, these lattice fringes belong to (111) Ge planes, with a lattice space of 0.326 nm. The average size of crystallized Ge-NPs is 3.8 ± 0.7 nm. The size of amorphous Ge-NPs appears to be bigger (5–6 nm) than of crystallized Ge-NPs as their interface with SiO_2_ matrix is not precise.

**Figure 3:**
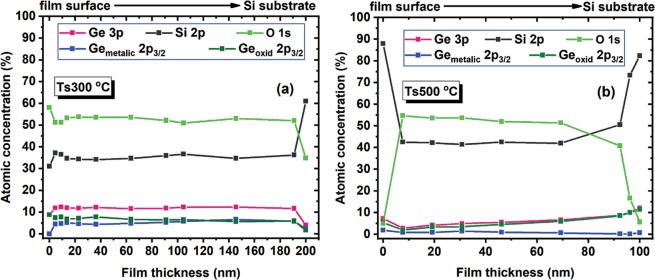
Atomic concentration dependence on the film thickness, for films deposited at (**a**) 300 °C and (**b**) 500 °C. The concentrations were obtained from XPS measurements, namely O 1 s, Ge 3p and Si 2p lines. The Ge and Si chemical states were obtained from Ge 2p_3/2_ and Si 2p XPS spectra.

In Fig. 3 are presented the atomic concentration depth profile results from XPS spectra measured on the film thickness, for films deposited at (a) 300 °C and (b) 500 °C after Ar^+^ etching (about 1 nm/min). These curves evidence the concentrations as a function of the film depth for metallic and oxidized Ge together with the total Ge concentration, oxygen concentration and also the Si concentration. One can see that all concentrations in the film deposited at 500 °C substrate temperature are smaller than those in the film at 300 °C, as the deposition rate at 500 °C is smaller. The concentrations for all specimens are constant along the film depth in sample deposited at 300 °C, while in the sample deposited at 500 °C the concentration of atomic Ge is low and varies a little in the film depth. In the sample deposited at 300 °C, Ge/Si ratio is 0.33 (total Ge concentration being 12–14%) is in good agreement with EDX data. The sample deposited at 500 °C is different, so almost whole amount of Ge is oxidized (the curve of oxidized Ge is very close to that of total Ge) and the concentration of oxidized Ge (together with total Ge) decreases from the film/Si interface to the free surface of the film. This suggests that a part of Ge is lost, even during film deposition at higher temperature by formation of GeO^36,38–40^ that is described by following reactions: (i) phase separation by *GeO*_*x*_ → *Ge* + *GeO*_2_ or by reduction of Ge oxides by Si or Si sub-oxides via *GeO*_*x*_ + *SiO*_*y*_ → *Ge* + *GeO*_*x*−*z*_ + *SiO*_*y*+*z*_ (this reaction starts at 300 °C) and then (ii) Ge loss by reaction $\frac{1}{2}\,$*GeO*_2_*(solid)* + $\frac{1}{2}\,$*Ge(solid)* → *GeO(gas)* (this becomes dominant at temperature over 400 °C^38,39,41^.

### Deposition temperature and illumination effects on dark electric current

All Ge-NPs:SiO_2_ films were characterized by measuring *J*–*V* curves in dark and under illumination with incandescent lamp.

**Figure 4:**
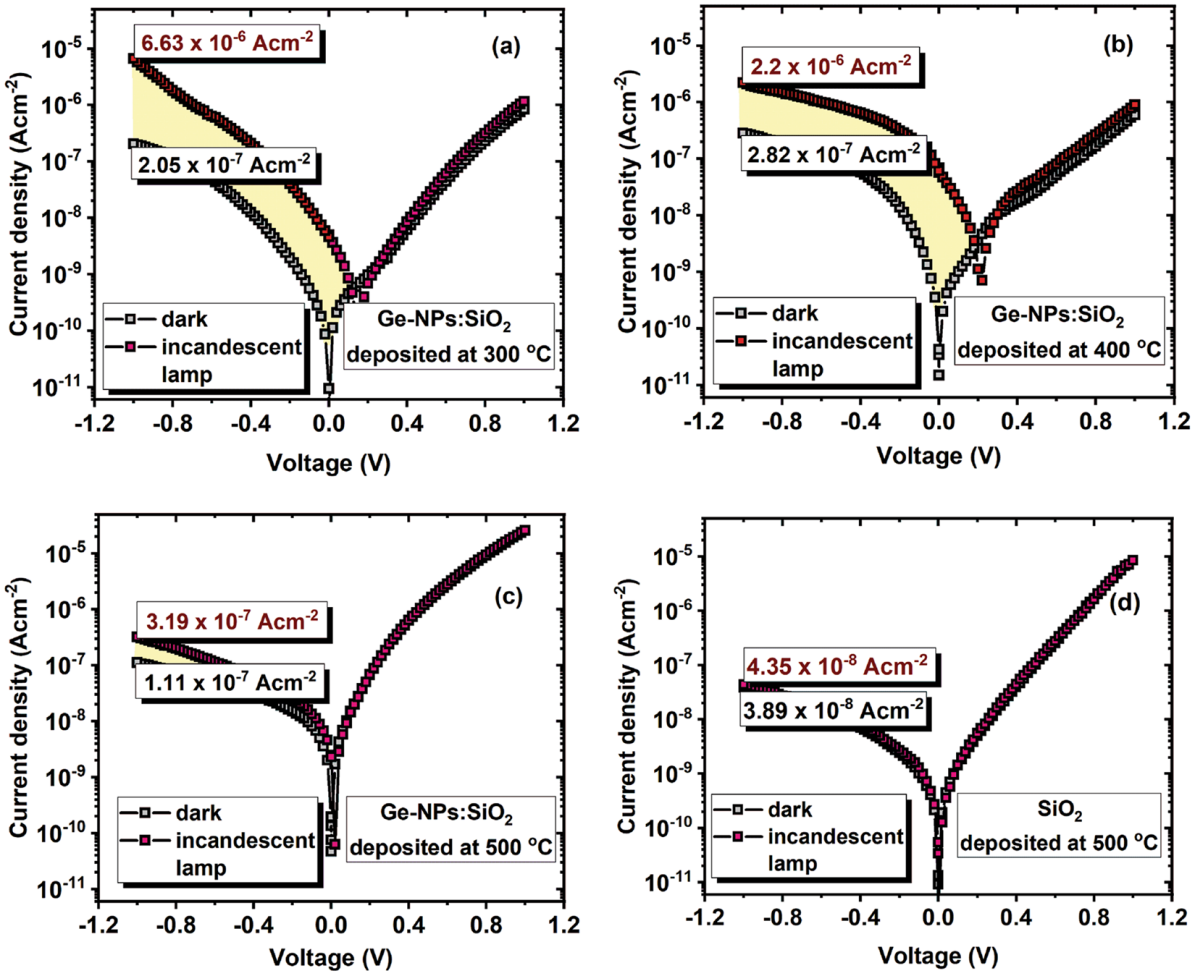
*J*–*V* curves taken at RT, in dark and under illumination with an incandescent lamp, on a Al/Si-n/Ge-NPs:SiO_2_/ITO structure annealed at: 300 °C (**a**), 400 °C (**b**) and 500 °C (**c**). Similar structure without Ge incorporated into the SiO_2_ layer deposited at 500 °C (**d**).

In Fig. 4 are shown *J*–*V* characteristics taken in dark and under illumination, at RT, on Ge-NPs:SiO_2_ thin films deposited on heated substrates at 300, 400 and 500 °C together with *J*–*V* characteristic measured on a SiO_2_ film deposited at 500 °C (without Ge). One can see that almost all *J*–*V* characteristics show a low rectifying behavior, while for 500 °C this shows a good diode behavior (Fig. 4c) mainly due to the Ge-NPs:SiO_2_ film/Si interface, according to XPS spectra. These *J*–*V* curves show that under illumination, the reverse photocurrents measured on films deposited at 300 and 400 °C are higher with at least one order of magnitude compared with dark currents, proving that these Ge-NPs:SiO_2_ films are highly photosensitive. These results are in good agreement with XPS results. Similar gains were reported in literature on different Ge-based films^1,42–44^. The *J*–*V* curves plotted in Fig. 4d show that the SiO_2_ layer without Ge-NPs embedded in is not photosensitive. Also, Ge-NPs:SiO_2_ films deposited at RT show a very poor photosensitivity.

### Deposition temperature effect on optical properties

**Figure 5:**
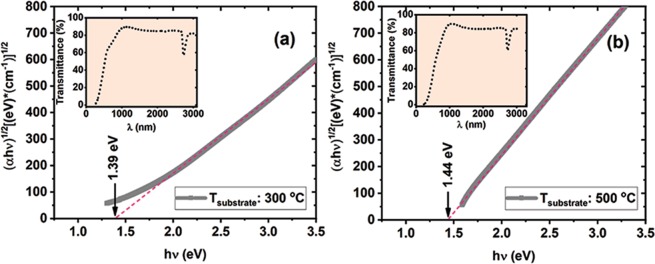
Absorption coefficient:–Tauc plot (solid lines) and linear fits (dash lines) for Ge-NPs:SiO_2_ deposited by heating the substrate at 300 °C (**a**) and 500 °C (**b**). Insets: corresponding transmittance spectra for 300 and 500 °C deposition temperature are represented.

The optical properties of the films deposited on quartz (Ge-NPs:SiO_2_/quartz sample) at 300 °C and 500 °C are presented in Fig. 5a, b. The curves of absorption coefficient *α*, in Tauc representation of (*α*hν)^1/2^ versus h *ν*, together with the transmittance T (insets) for each film deposited at 300 and 500 °C are given, considering the contribution of quartz substrate (T_*Q*_).

The absorption coefficient was obtained considering formula^45^:

1 $$\alpha =\frac{1}{d}ln\frac{{T}_{Q}(1-{R}_{S})}{{T}_{S}}$$

where d is the film thickness, T_*Q*_ is the transmittance of the quartz substrate, R_*S*_ is the spectral reflectance of the sample and T_*S*_ is its transmittance. An estimation of the optical band gap E_*g*_ was made by representing the absorption coefficient *α* in Tauc representation (Fig. 5a,b)^46^. The corresponding optical bandgaps are E_*g*_ = 1.39 eV for sample deposited at 300 °C and E_*g*_ = 1.44 eV for sample deposited at 500 °C. The higher optical E_*g*_ of 1.44 eV for 500 °C deposited sample can be explained by the lower Ge concentration and higher oxidation level in respect to that for 300 °C deposited samples (according to XPS results).

### Deposition temperature and illumination influence on spectral photocurrent

**Figure 6:**
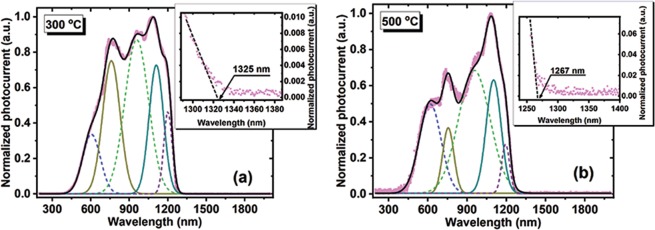
Photocurrent spectra obtained on films deposited at 300 and 500 °C substrate temperature. The insets present in detail the cutoff wavelengths.

Figure 6a,b present the photocurrent spectra that were normalized to maximum and obtained on Ge-NPs:SiO_2_ films deposited at 300 and 500 °C together with the deconvolution maxima.

**Figure 7:**
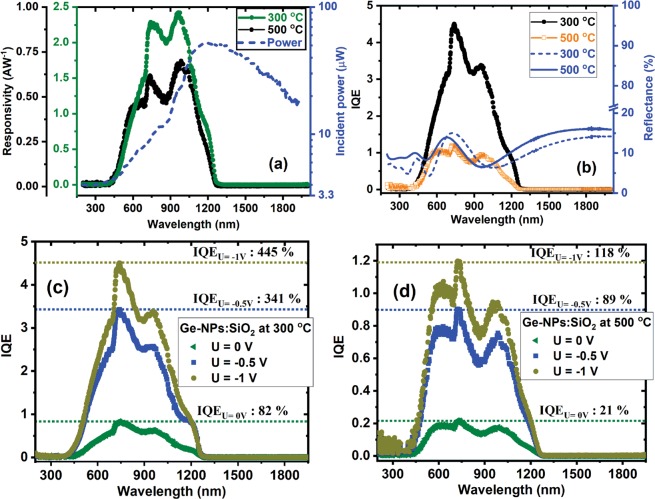
Ge-NPs:SiO_2_ films deposited at 300 and 500 °C: (**a**) Spectral responsivity (left axes) and incident power (right axes); (**b**) IQE (left axes) and reflectance (right axes); (**c**) and (**d**) Spectral IQE at different external applied voltages.

These characteristics were obtained for −1V reverse bias by using modulated light (80 Hz). One can observe that the Ge-NPs:SiO_2_ films have a broadband of sensitivity from 400 nm to 1325 nm. The cutoff wavelengths in the photocurrent spectra (insets in Fig. 6a,b) are ~1325 and ~126  nm for films deposited at 300 and 500 °C, respectively, that are higher than the wavelength edge corresponding to Si (~1100 nm). Also, with the deposition temperature increase, the cutoff wavelength shifts to higher energy. Similar results were obtained in ref.^47^. The photocurrent spectra present maxima and shoulders positioned at about the same wavelengths. These spectra are deconvoluted by five maxima positioned at about similar wavelengths (for films deposited at 300 and 500 °C substrate temperature) with different relative intensities depending on the deposition temperature. From the deconvolution of normalized current curves in Fig. 6a,b around 1100 nm and at longer wavelengths it results two peaks located at ~1100 nm and ~1200 nm, respectively. The (deconvoluted) maximum positioned at ~1100 nm is due to Si substrate^48^ and the peak at ~1200 nm can be attributed to Ge rich (with Ge-NPs) layer at the interface with Si substrate (Fig. 1). The other maxima and shoulders situated below 1100 nm can be attributed to photo-effects in Ge-NPs:SiO_2_ films, the main contribution being due to Ge related defects (most of them located at Ge-NP/SiO_2_ interface) acting as traps. Ge loss is increased in sample deposited at 500 °C according with XPS and absorption data. The responsivity spectra plotted in Fig. 7a (left axes) are calculated by using the equation:

2 $${R}_{\lambda }=\frac{{I}_{photo}(\lambda )}{{P}_{incident}(\lambda )}$$

where *I*_*photo*_ is the photocurrent measured under monochromatic light and *P*_*incident*_ is the incident optical power plotted in Fig. 7a (right axes). One can see that our films present good responsivities of 2.42 AW^−1^ for 300 °C and 0.69 AW^−1^ for samples deposited at 500 °C. With the increase of the substrate temperature from 300 to 500 °C the responsivity decreases with about four times, probably due to Ge loss and oxidation. We also calculated the internal quantum efficiency (IQE) presented in Fig. 7b (left axes) by using the reflectance spectra obtained on Ge-NPs:SiO_2_ films deposited at 300 and 500 °C (Fig. 7b (right axes)), by using the equation:

3 $$IQE=\frac{hc}{q\lambda (1-R)}\ast {R}_{\lambda }$$

where h is Planck constant, c–the speed of light in vacuum, q–the elementary charge, *λ*–the wavelength, R–the reflectance and R_*λ*_–spectral responsivity. It can be observed that IQE maximum value is 445% for the film deposited at 300 °C and a smaller value of about 118% for 500 °C deposited film.

The high internal IQE in our samples can been explained by considering trapping of the photogenerated holes on Ge related defects/traps. Higher than 100% quantum efficiency is also reported by other groups^3,42–44^.

The photoholes are generated by local photon absorption in the layer, but also can be due to injection of photoholes from Si substrate. The combined effects, those of the trapping of photoholes and electron extraction by electric field into more extended states result in equivalent increase of the electron lifetime and high photosensitivity dependent on electric field. The photogenerated electrons with Δn density moves through the film repetitively during their longer lifetime if the time of flight is shorter than their lifetime. The photocurrent (*I*_*photo*_) in given by the equation:

4 $${I}_{photo}=q{\rm{\Delta }}n\mu E$$

in which q- the elementary charge, *μ*- the mobility, E–the electric field, Δn = Φ*ητ*, where Φ–is the incident photons flux, *η*–effective photogeneration efficiency, and *τ*–effective lifetime.

For supporting this explanation, in Fig. 7c,d are presented the IQE spectra obtained for different external applied voltage 0, −0.5 and −1 V. Other mechanisms reported in literature that can explain higher that 100% internal efficiency are related to the reduction of the junction barrier by hole trapping, also observed within other materials as for example in GaN photodetectors^49^. The classical carrier multiplication present in avalanche photodiodes is unlikely for such disordered materials with poor mobility of carriers.

Therefore, the samples with high performance parameters are the 300 °C and 400 °C deposited films in which Ge content is uniform distributed in the depth. The films of Ge-NPs:SiO_2_ are photosensitive materials in a broadband whose spectral response can be tuned by adjusting the substrate temperatures during magnetron sputtering deposition. This makes Ge-NPs:SiO_2_ films deposited at 300 °C and 400 °C to be proper materials for optical sensors application having up to 445% IQE.

An additional advantage of Ge-NPs:SiO_2_ films for optical sensors application is that they present a relatively low dark current, leading to a good signal-to-noise ratio and device consumption^1,19^. The performant characteristics we obtained in our samples for VIS-NIR can be easily used for fabrication of high sensitive optical sensors on Si wafers and flexible substrates, due to low temperatures used during deposition.

## Conclusions

In this work, we fabricated and investigated films of Ge-NPs:SiO_2_ that are photosensitive in a VIS-NIR broadband (400–1325 nm). The films are deposited by magnetron sputtering on heated Si and quartz substrates maintained at different temperatures of 300, 400, 500 °C. The morphology of the films is quite similar, the films being formed of amorphous Ge-NPs:SiO_2_. In the films deposited at 500 °C substrate temperature few crystalized Ge-NPs were observed, the majority of them remaining amorphous. In the films deposited at 300 °C, the Ge content is constant in the film depth, while the films deposited at 500 °C show a significant decrease of Ge content from the interface of the film with substrate towards the film free surface. This Ge depletion observed at the free surface is related to Ge oxidation, and consequently Ge loss via GeO. The *J*–*V* characteristics measured on all films show that photocurrent densities are higher than the dark ones, being with more than one order of magnitude for 300 and 400 °C deposition temperature. The optical absorption measured on films deposited at 300 °C evidences a band gap E_*g*_ = 1.39 eV while in the films deposited at 500 °C, E_*g*_ = 1.44 eV was found. The photocurrent spectra present different cutoff wavelengths of ~1325 nm for films deposited at 300 °C and ~1267 nm for those at 500 °C, this shift to higher energy being due to oxidation and consequently Ge loss. The photocurrent spectra present maxima and shoulders positioned at about the same wavelengths. These spectra are deconvoluted by five maxima positioned at about similar wavelengths with different relative intensities depending on the deposition temperature. The maximum positioned at about 1100 nm obtained by deconvolution is due to Si substrate contribution. The peak positioned at ~ 1200 nm (from deconvolution) can be attributed to Ge rich (with Ge-NPs) layer at the interface with Si substrate. The other maxima and shoulders situated below 1100 nm can be attributed to photo-effects in Ge-NPs:SiO_2_ films, the main contribution being due to Ge related defects (most of them located at Ge-NP/SiO_2_ interface) acting as traps. The films deposited at 300 and 500 °C have good responsivities (2.42 AW^−1^ for samples deposited at 300 °C and at 0.69 AW^−1^ for samples deposited at 500 °C) and IQE (~445% for 300 °C and ~118% for 500 °C). We demonstrated that the films of Ge-NPs:SiO_2_ obtained by heating the substrate at 300 °C and 400 °C present the high sensitivity, responsivity and IQE values, that make them suitable for photodetectors application in NIR due to the possibility of tuning the sensitivity and the spectral range.

## Methods

### Preparation of Ge-NPs:SiO_2_ thin films

**Figure 8:**
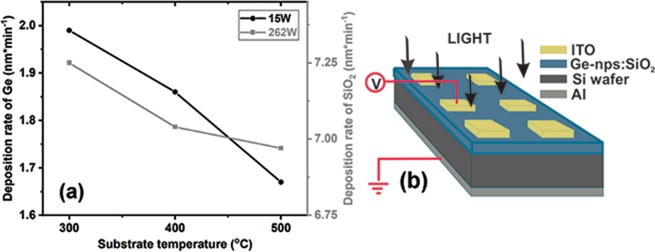
(**a**) Deposition rates at different substrate temperatures for Ge (left axes) and SiO_2_ (right axes). (**b**) The sketch of a sample with a top–down configuration; schematic of the measurement setup.

Amorphous Ge-NPs:SiO_2_ thin films were deposited by using a Surrey Nanosystems Gamma1000 magnetron sputtering system equipped with multi-target assembly. As substrates, we have used quartz and (100) n-type Si with resistivity in the range 10–20 Ωcm. The film deposition has been made on heated substrates at three different temperatures of 300, 400 and 500 °C, respectively. For this, Ge and SiO_2_ were simultaneously deposited from separate targets, controlling technological parameters (Ar atmosphere, work pressure of 4 mTorr, substrate temperature and applied power on each target) and consequently the film thickness, uniformity and composition. Figure 8a presents the deposition rates of Ge and SiO_2_ at 300, 400 and 500 °C substrate temperatures.

We have established the deposition rates by depositing films of Ge and SiO_2_ respectively, at different substrate temperatures. These films were investigated by ellipsometry (Woollam M-2000) for obtaining the deposition rates. The deposition rate during sputtering decreases with the temperature increase mainly due to the reflection of species on the substrate. Also, at high temperature we have to consider the formation of GeO gas that will be lost (in Fig. 3(b) oxygen concentration is very low at the free surface of the film), the mobility of species at 500 °C being increased^41,50^.

### Characterization

For electrical and photo-electrical measurements transparent Indium-Tin-Oxide (ITO) contacts of 3 × 3 mm^2^ were deposited on the top side of samples using a magnetron sputtering tool (Varian model ER3119) and Al contacts on the back side of Si wafers by e-beam evaporation (Bestec). Figure 8b shows a sketch of samples with configuration (Al/Si-n/Ge-NPs:SiO_2_ film/ITO) used for (photo)electrical investigations. The morphology of films was investigated by using X-ray Photoelectron Spectroscopy (XPS) (SPECS equipment together with a PHOIBOS 150 analyzer) and Cross Section Transmission Electron Microscopy (XTEM) using a JEOL analytical atomic microscope (JEM ARM 200F). For electrical and photoelectrical investigations was used an experimental setup formed by a closed cycle cryostat (Janis CCS-450), a Keithley electrometer (6517A) with an internal voltage source, a 331 LakeShore controller for controlling the measurement temperature and a tungsten-halogen lamp (20 W, Newport) as light source. Spectral measurements were done using a tungsten-halogen light source (70 W) mounted in the setup together with a 1/4 Newport monochromator (200–2500 nm), a Stanford Research light modulator (SR540) and a double lock-in amplifier (SR830). The incident optical power was measured using a Thorlabs power meter PM100D equipped with S401C detector. Reflectance spectra were measured at fixed incident angle (6°) using a Perkin Elmer 950 UV-VIS-NIR spectrophotometer equipped with specular reflectance accessory (B0086703).
